# Cold Plasma Treatment Increases Bioactive Metabolites in Oat (*Avena sativa* L.) Sprouts and Enhances *In Vitro* Osteogenic Activity of their Extracts

**DOI:** 10.1007/s11130-022-01029-3

**Published:** 2022-11-16

**Authors:** Mi Ja Lee, Hyun-Jin Lee, Yongjin Lee, Ji Yeong Yang, Jong Seok Song, So Yeun Woo, Hyun Young Kim, Seung-Yeob Song, Woo Duck Seo, Young-Jin Son, Sung Il Park

**Affiliations:** 1 Division of Crop Foundation, National Institute of Crop Science (NICS), Rural Development Administration (RDA), Wanju, 55365 Korea; 2 Department of Pharmacy, Sunchon National University, Suncheon, 57922 Republic of Korea; 3 Institute of Plasma Technology, Korea Institute of Fusion Energy (KEF), Gunsan, 54004 Korea; 4 Korea Research Institute of Bioscience & Biotechnology, Cheongju, 28116 Korea

**Keywords:** Oat sprout, Phytochemical, Plasma, GABA, Osteoblast differentiation

## Abstract

Cold plasma treatment has been studied to enhance the germination, growth, and bioactive phytochemical production in crops. Here, we aimed to investigate the effects of cold plasma treatment on the growth, bioactive metabolite production, and protein expression related to the physiological and osteogenic activities of oat sprouts. Oat seeds were soaked for 12 h, and then exposed to plasma for 6 min/day for 3 days after sowing. Plasma exposure did not significantly change the growth of oat sprouts; however, increased the content of bioactive metabolites. A single exposure for 6 min on the first day (T-1) increased the content of free amino acids (39.4%), *γ*-aminobutyric acid (53%), and avenacoside B (23%) compared to the control. Hexacosanol content was the highest in T-3 (6 min exposure on each day for 3 days), 28% higher than that in the control. Oat sprout extracts induced the phosphorylation of adenosine 5′-monophosphate-activated protein kinase and osteoblast differentiation was enhanced by increasing the alkaline phosphatase (ALP) activity; all these effects were induced by plasma treatment. Avenacoside B content was positively correlated with ALP activity (*r* = 0.911, *p* < 0.1). These results suggest that plasma treatment has the potential to improve the value of oat sprouts and that it may be used in food fortification to enhance nutritional value for promoting human health.

**Supplementary Information:**

The online version contains supplementary material available at 10.1007/s11130-022-01029-3.

## Introduction

Consumer awareness of a healthy lifestyle in pursuit of health and longevity has driven interest in functional food with health benefits. Health-conscious people are choosing plants with high content of functional ingredients because synthetic material use is being restricted due to the associated toxicity [1]. Plant-derived bioactive molecules, called phytochemicals (*e.g*., phenolic, saponin, triterpenoid, and flavonoid), have diverse pharmacological properties such as anticancer, antidiabetic, anti-oxidant, and anti-atherosclerotic activities. Plants are one of the food sources providing commercial chemicals and specialized metabolites to humans and other animals. Thus, there is growing interest in producing natural food sources with high content of nutrients and bioactive compounds without reducing production [2, 3]. Sprouts have emerged as functional foods because of their high nutrient (vitamins, minerals, and amino acids) and phytochemical (phenolics and flavonoids) content [4]. Studies have reported barley, oat, and wheat sprouts as good functional food sources because they contain health-promoting compounds such as polyphenols, saponarin, *γ*-aminobutyric acid (GABA), and policosanols [5]. Oat (*Avena sativa* L.) is among the healthiest foods, rich in fiber, fatty acids, and proteins. It contains phytochemicals such as steroidal saponins, avenanthramides (AVAs), phenolic acids, tocols, and flavonoids, that benefit human health; certain saponins accumulate in sprouts rather than seeds [6]. Oat sprouts prevent bone disorders [7], and wheat sprouts help alleviate obesity and diabetes [4]. To enhance germination and growth of and increase bioactive compound content in sprouts, physical and chemical methods [8] and application of LED lights and cold plasma have been evaluated as pretreatment methods in the field of agricultural science [9, 10]. Plasma is a partially ionized gas containing electrons, photons, atoms, radicals, and excited and ground-state molecules with a net neutral charge. Recently, the use of plasma treatment has been studied as a tool for stress-induced modification and intensification of the production of secondary metabolites in plants [10]. The use of plasma enhances seed germination, promotes crop growth, and affects metabolic processes, including secondary metabolite biosynthesis [11]. However, information on the effects of plasma on growth, bioactive phytochemicals, and the associated enzyme and protein expression in oat sprouts is limited. Additionally, there have been only a few studies on the bioactivity of plasma-treated oat sprouts. We hypothesized that plasma treatment during germination would act as an external stress and change the primary metabolites such as amino acids and increase the content of phytochemicals according to plasma treatment conditions. The objective of this study was to examine the effects of plasma treatment on oat sprouts and determine whether this plasma treatment is useful in the production of bioactive metabolites in oat sprouts.

## Materials and Methods

### Plant Growing Conditions and Preparation of Oat Sprout Extract

*Avena sativa* ‘Daeyang’ cultivar used in this study is a naked oat cultivar developed in South Korea. It was harvested at the National Institute of Crop Science, Rural Development Administration, South Korea, in 2020. Germination of oat seeds was evaluated by placing 100 randomly selected seeds on two Whatman No. 40 filter papers in 9-cm petri dishes containing 15 mL distilled water. Three replicates were prepared. The covered petri dishes were placed in an incubator in the dark at 17 ± 1 °C. Germination counts were made after six days of incubation.

$$\mathrm{Germination}\;\mathrm{percentage}\hspace{0.17em}=\hspace{0.17em}\mathrm{number}\;\mathrm{of}\;\mathrm{germinated}\;\mathrm{seeds}/\mathrm{number}\;\mathrm{of}\;\mathrm{total}\;\mathrm{seeds}\hspace{0.17em}\times\hspace{0.17em}100$$

For growing oat sprouts, 20 g of oat seeds were soaked for 12 h in water and grown for 9 days in a growth chamber at 17 °C with a 16/8 h light/dark cycle and 50% humidity, which was maintained by regular sub-irrigation. The growth period was set at 9 days because there was no significant change in growth, and leaf tips dried after this period. On day nine, leaf length was measured, and the leaves were collected to measure fresh weight. Collected leaves were lyophilized and ground using a Retsch centrifugal mill (Zm 100, Retsch GmbH, Haan, Germany) with a 0.2-mm sieve, and then stored in a 4 °C chamber in all experiments. Oat sprout flour was extracted with ethanol for 24 h at 20 °C, and filtered through Whatman No. 3 (Whatman, Maidstone, UK) filter paper. Thereafter, the plant extract was concentrated in a rotary evaporator (EYELA Co., Ltd., Shanghai, China) to produce oat sprout extract (OSE).

### Plasma Treatment

Plasma treatment was performed using a prototype acrylic chamber of 30 L volume, equipped with surface dielectric barrier discharge (SDBD) electrodes (National Fusion Research Institute, South Korea) [11]. The frequency, peak-to-peak voltage, and average power were 14.4 kHz, 8 kVp, and 51.7 W, respectively. The applied voltage was measured on the upper electrode connected to a power supply using a 1000:1 high voltage probe (P6015A, Tektronix, Beaverton, OR, USA), and discharge current on the grounded lower electrode using a current probe (110A, Pearson Electronics, Palo Alto, CA, USA). Then, the traces of the applied voltage and discharge current were recorded on an oscilloscope (DPO4104B-L, Tektronix). An optical emission spectrum was measured using an optical emission spectroscope (HR4000CG-UV-NIR, Ocean Insight, Orlando, FL, USA). Concentrations of gas-phase ozone and nitrogen oxides were measured using an ozone analyzer (GM-PRO, Anseros, Tübingen, Germany) and a NO_X_ analyzer (T200, Teledyne, San Diego, CA, USA). The maximum ozone concentration was 130 *μ*L/L, and the nitrogen oxide concentration in the chamber was 0.8 *μ*L/L.

Plasma treatment was performed only for three days after sowing. Treatment time was 6 min/day, and 20 g of oat seeds was treated. T-con indicates the control without plasma treatment. T-1 indicates single treatment with 6-min exposure on day 1. T-2 indicates double treatment with 6-min exposure on days 1 and 2. T-3 indicates triple treatment with 6-min exposure on days 1, 2, and 3. Because there were no significant changes in growth and leaf tip dried out after 9 days, the growth period was set to 9 days. The distance between the SDBD electrode and the sample dish was 30 cm. Oat samples were treated in triplicate.

### Determination of the Amino Acid and GABA Contents

Amino acid and GABA contents were determined using the AccQ Tag method (Waters, Milford, MA, USA) and an ultra-performance liquid chromatography (UPLC) system equipped with a 2475 multi fluorescent detector (Waters) according to the method of Song et al. [11]. The total amino acid content was calculated as the sum of the analyzed amino acids.

### Determination of Total Phenolic and Policosanol Content

Total phenolic content was determined using the Folin–Ciocalteu method [12]. The sample was extracted using 80% MeOH, and the absorbance was measured at 720 nm. The policosanol content was analyzed using an Agilent 7890A gas chromatography system coupled with a 5977A single quadrupole mass spectrometer (Agilent Technologies, Palo Alto, CA, USA) [4].

### Determination of Steroidal Saponin Content

To analyze steroidal saponin content, oat sprout powder (0.1 g) was extracted using 20 mL of hexane for 16 h at 25 °C. The extract was centrifuged at 15,000 × *g* for 10 min, and the supernatant was evaporated to dryness in a rotary evaporator (EYELA N-1000, Keyland Court Bohemia, NY, USA). The crude extract was re-extracted with 20 mL of MeOH for 5 h and filtered through a 0.2-*μ*m RC syringe filter. Steroidal saponin content was determined using a UPLC–ELSD (Waters) system [6, 7]. The column was a Halo C-18 column (2.7 *μ*m, 100 mm × 2.1 mm inner diameter), the temperature was 35 °C, and the solvents were mobile phase A (0.1% formic acid in distilled water, v/v) and mobile phase B (100% acetonitrile).

### Analysis of mRNA Expression in Oat Sprouts

Oat sprouts were ground in liquid nitrogen in a mortar, and extracted with TRIzol reagent (Invitrogen, Carlsbad, CA, USA). The PCR primer sets were designed using the online Primer3 program (Teitelbaum, 2000). The PCR primer sequences used were as follows: *HMGCR*, sense 5′- TGTCCCCACTATGACTTCCC-3′, anti-sense 5′-TCGGTGGCCTCTAGTGAGAT-3′; *GAD65*, sense 5′-CTGCTCCAGTCTCCAAAGCC-3′, anti-sense 5′-CCGTGAACTTCTG AGCCACT-3′; *β‐Actin*, sense 5′-TCACCCACACTGTGCCCATCTACGA-3′, anti-sense 5′-CAGCGGAACCGCTCATTGCCAATGG-3′. The total RNA was isolated, and 1 μg of RNA was reverse transcribed using the M-MLV cDNA Synthesis kit (Enzynomics, Daejeon, Korea), according to the manufacturer’s protocol. Quantitative PCR was performed using the TOPreal qPCR 2 × PreMIX (Enzynomics) and Real-Time PCR detection system (Bio-Rad, Hercules, CA, USA). All tests were performed in triplicate, and the expression levels were normalized to the level of the housekeeping gene *GAPDH*.

### Analysis of Protein Expression in Oat Sprouts

Oat sprouts were ground in liquid nitrogen with a mortar, and extracted with lysis buffer containing 20 mM Tris–HCl of pH 7.5, 1% v/v Igepal CA-630, 150 mM NaCl, 1 mM sodium fluoride (Sigma–Aldrich, St. Louis, MO, USA), 1 mM sodium orthovanadate (Sigma–Aldrich), 0.5 mM phenylmethylsulfonyl fluoride (Sigma–Aldrich), 1 mM DTT, 2 mM EDTA, 10 μg/mL Aprotinin (Sigma–Aldrich), 5 *µ*g/mL leupeptin (Sigma–Aldrich), and 2 *µ*g/mL pepstatin (Sigma–Aldrich). The lysates were centrifuged at 12,000 × *g* for 15 min at 4 °C. The protein extracts were subjected to sodium dodecyl sulfate polyacrylamide gel electrophoresis (SDS-PAGE) and transferred onto polyvinylidene difluoride membranes (Millipore, Billerica, MA, USA). The membranes were blocked for 1 h in Tris-buffered saline containing 0.1% Tween 20 (TTBS) and 5% skim milk. Immunoblotting was conducted by overnight incubation; the membranes were incubated with secondary antibodies conjugated to horseradish peroxide for 3 h at 20 °C. After washing, the bands were viewed using a luminescent image analyzer MicroChemi 4.2 (DNR Bio-Imaging System, Jerusalem, Israel).

### Osteoblast Differentiation

All osteoblast-mediated experiments were performed as previously described [13]. Mouse mesenchymal precursor C2C12 cells from the American Type Culture Collection (Manassas, VA, USA) were maintained in alpha minimal essential medium (*α*-MEM) containing 10% FBS, 100 unit/mL penicillin, and 100 *µ*g/mL streptomycin. The cells were seeded at 2.5 × 10^3^ cells/well in 96-well plates or 2.5 × 10^5^ cells/well in 6-well plates. After 1 day, the cells were cultured in *α*-MEM containing 5% FBS and rhBMP-2 (100 ng/mL). The cells were differentiated by replacing the medium with 5 *µ*g/mL OSE. Osteoblastic bone formation was observed using alkaline phosphatase (ALP) staining.

### Alkaline Phosphatase Staining and Activity Assays

For staining ALP, an initial biomarker for osteoblast differentiation, the cells were differentiated for 3 days, washed twice with phosphate buffered saline (PBS), fixed with 10% formalin in PBS for 5 min, rinsed with deionized water, and stained using the ALP Kit (Sigma–Aldrich). To measure ALP activity, the differentiated cells were washed twice with PBS, fixed with 10% formalin in PBS for 5 min, rinsed with PBS, and then washed with one-step PNPP 50 *μ*l/well substrate solution (Thermo Scientific, Waltham, MA, USA), according to the manufacturer's instructions. After measuring at 405 nm every 5 min, it terminates when the activity begins to decrease.

### Statistical Analysis

All statistical analyses were conducted using SAS 7.0 (SAS Institute Inc., Cary, NC, USA). Data were assessed using the analysis of variance, and a mean comparison was performed using Duncan’s multiple range test at *p* < 0.05. Statistical differences in ALP activity were analyzed using Student’s *t-*test, and results with *p* < 0.05 were considered statistically significant.

## Results and Discussion

### Effect of Plasma Treatment on the Growth of Oat Sprouts

**Fig. 1:**
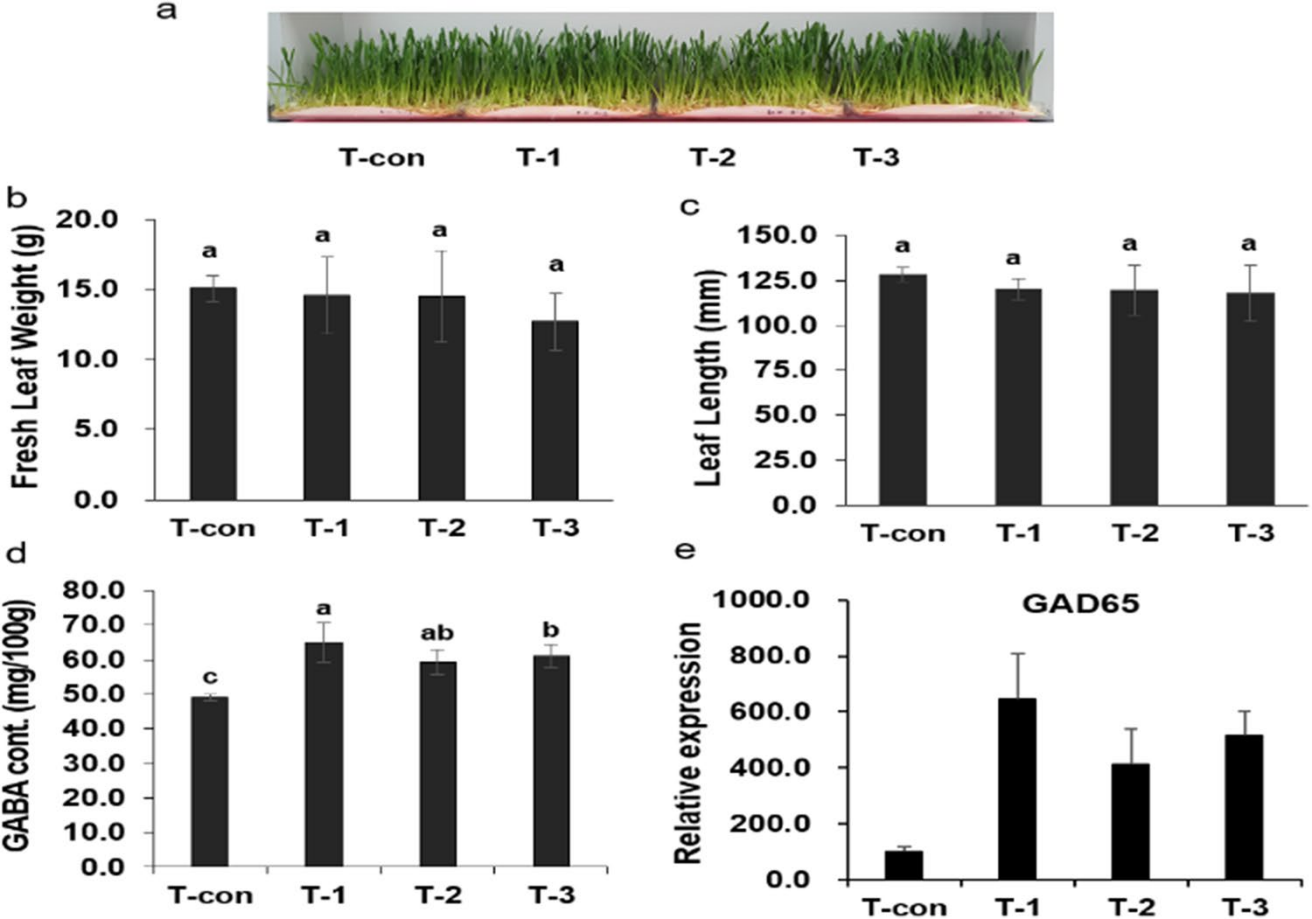
Appearance (**a**), fresh leaf weight (**b**), leaf length (**c**), *γ*-Aminobutyric acid (GABA) content (**d**) and mRNA expression of GAD65 (**e**) of oat sprouts at 9 days after sowing, each exposed to plasma for 6 min/day and ventilated as follows: T-con (the control without plasma treatment), T-1 (6 min exposure for 1 day), T-2 (6 min exposure on each day for 2 days), T-3 (6 min exposure on each day for 3 days). Each bar represents the mean of three replicates. Means with the same letter are not significantly different according to Duncan’s multiple range test (DMRT) at *p* < 0.05

The germination percentage of the oat seeds observed in this research was 88.2%. The growth characteristics of sprouts grown for nine days after sowing and plasma exposure for early growth assessment are shown in Fig. 1. The fresh weight of leaves ranged from 12.7 to 15.1 g (Fig. 1b). The leaf length was 111.9–129.4 mm (Fig. 1c). The plasma treatment conditions had no significant effect on the growth of oat sprouts. Plasma can increase seedling growth because of the production of reactive nitrogen species by discharge [10, 11]. Contrarily, excessive plasma exposure substantially inhibits seedling growth [14], possibly owing to its phytotoxic effects such as induced ozone phytotoxicity and the acidity of plasma-treated water on the seedlings. Therefore, optimizing the seed plasma treatment time and treatment conditions for each type of seed is crucial [15].

### Amino Acid Content in Oat Sprouts

Amino acids are directly or indirectly involved in the regulation of plant responses to various stresses and environmental signals (light, and biotic and abiotic stresses) [3]. A general accumulation of free amino acids has usually been observed in different plants exposed to abiotic stress and this accumulation might be different for individual amino acids. Aromatic amino acids (phenylalanine, tyrosine, and tryptophan) are components of protein synthesis and precursors of secondary metabolites. Here, we analyzed amino acid content to investigate the effect of plasma exposure on the amino acid metabolism in oat sprouts (Table S1). The total amino acid content was the highest in T-1 and there was no significant difference from the control in other treatment conditions. The content of arginine, glutamic acid, and proline, which are related to stress regulation in the amino acid metabolism in plants, was increased by 46.6, 86.0, and 49.5%, respectively. The content of the essential amino acids threonine, lysine, and methionine increased by 58.8, 145.4, and 58.6%, respectively. The content of the aromatic amino acids tyrosine and phenylalanine increased by 17.3 and 14.8%, respectively. Additionally, the isoleucine and cysteine content increased by 74.5 and 62.7%, respectively. Under the plasma treatment conditions used in this study, reactive nitrogen species in the water and chamber may enhance nitrogen metabolism in plants [11, 15].

### Content of GABA and Gene Expression of Glutamic Acid Decarboxylase 65 in Oat Sprouts

GABA is known as a stress-responsive metabolite produced through the enzymatic decarboxylation of glutamic acid. Here, the GABA content in all plasma-treated samples was higher than that in T-con (Fig. 1d). A similar result for SDBD exposure and its effect has been reported for barley sprouts, which showed an increase in GABA content [16]. T-1 showed the highest GABA content, 53% higher than that in the control. The GABA content in T-2 and T-3 increased by 32 and 12.2%, respectively, compared with that in the control. The increase in GABA content may be associated with increasing GABA shunt-related metabolites such as glutamic acid and alanine in oat sprouts after plasma exposure [11]. Here, glutamic acid and alanine content in T-1 increased 86.0 and 48.9%, respectively (Table S1). It is known that glutamate is converted into GABA by L-glutamic acid decarboxylase *in vivo*. GAD65 is responsible for catalyzing the production of GABA from L-glutamic acid [17]. Therefore, conditions in which GABA expression is increased can be selected through real-time qPCR analysis after plasma treatment. Here, the mRNA expression of GAD65 increased in T-1, T-2, and T-3 than compared to that of T-con (Fig. 1e).

### Total Phenol and Policosanol Content in Oat Sprouts

The effect of plasma treatment on the content of phytochemicals was investigated. The total polyphenol content (TPC) in T-3 was 8.5% higher than that in T-con (Table 1). TPC exhibited no clear trend in relation to plasma treatment condition. Yodpitak et al. [18] reported that TPC content of brown rice increased by DBD exposure during germination, whereas TPC of untreated and plasma treated rice increased at different rates. TPC of plasma treated rice reached its maximum within 0.5 days, and that of the control reached a maximum after 1.5 days of germination, and then rapidly decreased thereafter [18]. Plasma treatment enhances the TPC in oat sprouts possibly by triggering metabolic processes including the antioxidant system [11]. However, after plasma treatment, bioactive phytochemicals showed different trends depending on the treatment condition [18]. Therefore, it is necessary to investigate the TPC change of oat sprouts during growth after plasma treatment in the future. The effect of plasma treatment on policosanol, an antioxidant and health-promoting aliphatic alcohol known as a lipid-lowering agent, was investigated (Table 1). As reported for barley sprouts [19], hexacosanol was the most abundant policosanol in oat sprouts. The hexacosanol content increased as the number of plasma treatments increased. Thus, T-3 led to the highest hexacosanol content, which was 28% higher than that in the control. The policosanol profiles and their content may be markedly influenced by genetic and environmental stresses [19]. Plasma exposure can also increase the policosanol content in oat sprouts.

**Table 1:** Total polyphenol, polycosanol and steroidal saponin content of oat sprouts at 9 day after planting with exposure to plasma for 6 min/day and ventilated as follows: T-con (the control without plasma treatment), T-1 (6 min exposure for 1 day), T-2 (6 min exposure on each day, for 2 days), and T-3 (6 min exposure on each day, for 3 days)

Sample	Total polyphenol content (%)	Polycosanol content (mg/100 g)			
		Hexacosanol	Octacosanol	Triacontanol	Total
T-con	0.589 ± 0.012^b^	369.2 ± 3.5^c^	56.1 ± 0.1	27.9 ± 0.0	453.2^c^
T-1	0.563 ± 0.006^bc^	376.7 ± 6.0^bc^	56.0 ± 0.1	27.9 ± 0.0	460.6^c^
T-2	0.558 ± 0.011^c^	410.5 ± 3.7^b^	56.2 ± 0.1	31.0 ± 5.9	497.8^b^
T-3	0.644 ± 0.022^a^	472.4 ± 4.9^a^	56.7 ± 0.0	28.0 ± 0.0	557.2^a^
Steroidal saponin content (mg/100 g)					
	Isovitexin-2″-o-arabinoside	Isoswertisin-2″-o-rhamnoside	Avenacoside B	Avenacoside A	26-degluco avenacoside B
T-con	523.0 ± 6.1^ab^	604.6 ± 15.6^b^	750.9 ± 23.2^c^	407.4 ± 6.3^c^	538.5 ± 16.0^b^
T-1	511.4 ± 11.8^b^	597.1 ± 8.7^b^	923.5 ± 3.4^a^	464.8 ± 14.3^a^	392.8 ± 3.8^d^
T-2	510.7 ± 9.6^b^	636.6 ± 1.9^a^	824.3 ± 43.7^b^	437.8 ± 6.5^b^	464.9 ± 6.5^c^
T-3	533.7 ± 12.1^a^	619.1 ± 16.1^ab^	631.7 ± 22.1^d^	404.3 ± 3.6^c^	644.7 ± 17.5^a^

Data represent the mean ± standard error of triplicate experiments. Means with different letters within the same column are significantly different according to Duncan’s multiple range test at *p* < 0.05

### Analysis of HMG-CoA Reductase and AMPK Phosphorylation Associated with Policosanol

**Fig. 2:**
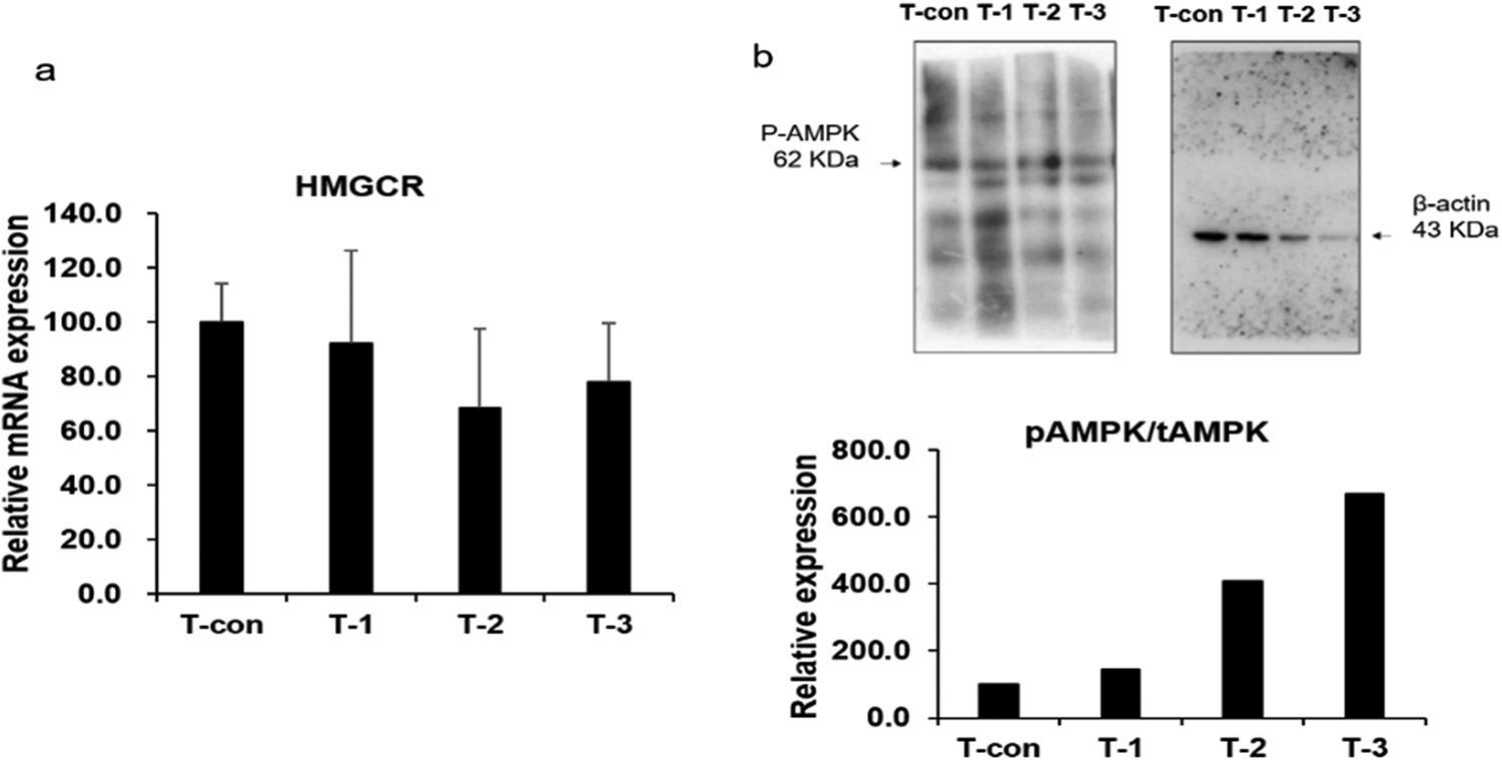
Inhibition of mRNA expression of *HMGCR* and increase in the phosphorylation of AMPK by policosanol. *HMGCR* was measured using real-time qPCR (**a**) and AMPK phosphorylation was investigated using western blotting (**b**). Each sprout was exposed to plasma for 6 min/day and ventilated as follows: T-con (the control without plasma treatment), T-1 (6 min exposure for 1 day), T-2 (6 min exposure on each day for 2 days), T-3 (6 min exposure on each day for 3 days)

*In vivo*, policosanol is an aliphatic alcohol [4, 19] containing carbon atoms and is found in natural sources, including beeswax and sugar cane. Its main effect is lowering the LDL cholesterol content and increasing the HDL cholesterol content. Additionally, policosanol inhibits the synthesis of HMG-CoA reductase (HMGCR) or promotes the degradation and induces the phosphorylation of AMP-activated protein kinase (AMPK) [19, 20]. We investigated the mRNA expression of *HMGCR* and phosphorylation of AMPK. Although not significant, *HMGCR* mRNA expression was inhibited by policosanol in the samples subjected to T-1, T-2, and T-3 (Fig. 2a), whereas the phosphorylation of AMPK was significantly increased (Fig. 2b). Ra et al. [4] reported that the policosanol content positively correlated with AMPK phosphorylation. Plasma treatment increased the content of policosanol and induced the activation of AMPK.

### Saponin Content in Oat Sprouts

Oat, the only saponin-accumulating cereal, contains two different forms of saponin: avenacosides and avenasides [14]. The accumulation of steroidal saponins in oats is associated with the growth of seedlings. Saponins are secondary metabolites, and they can be generated in response to external factors such as various biotic and abiotic stimuli [6, 15]. Table 1 shows the changes in the saponin content in oat sprouts after plasma treatment. The avenacoside A and avenacoside B content in oat sprouts was 4.0–4.6 and 6.3–9.2 g/kg DM, respectively. The UPLC chromatograms of the steroidal saponin standard solution and OSE are shown in Fig. S1. Recently, Woo et al. [7] isolated 10 steroidal saponins from oat seedlings and reported their activity in osteoblast differentiation. Here, only five of the sterols were detected (Table 1). The isoswertisin-2″-O-rhamnoside content in T-2 was 5.3% higher than that in T-con. Avenacoside B was the most abundant compound, with a 23% higher content in T-1 than in the control. The avenacoside A content was 14.1% higher in T-1, and the 26-deglucoavenacoside content was 19.7% higher in T-3 than in the control. Post-harvest plasma treatment of plant material enhanced the secondary metabolites in plants.

### OSE Enhances BMP-2-Mediated Osteoblast Differentiation in C2C12 Cells

**Fig. 3:**
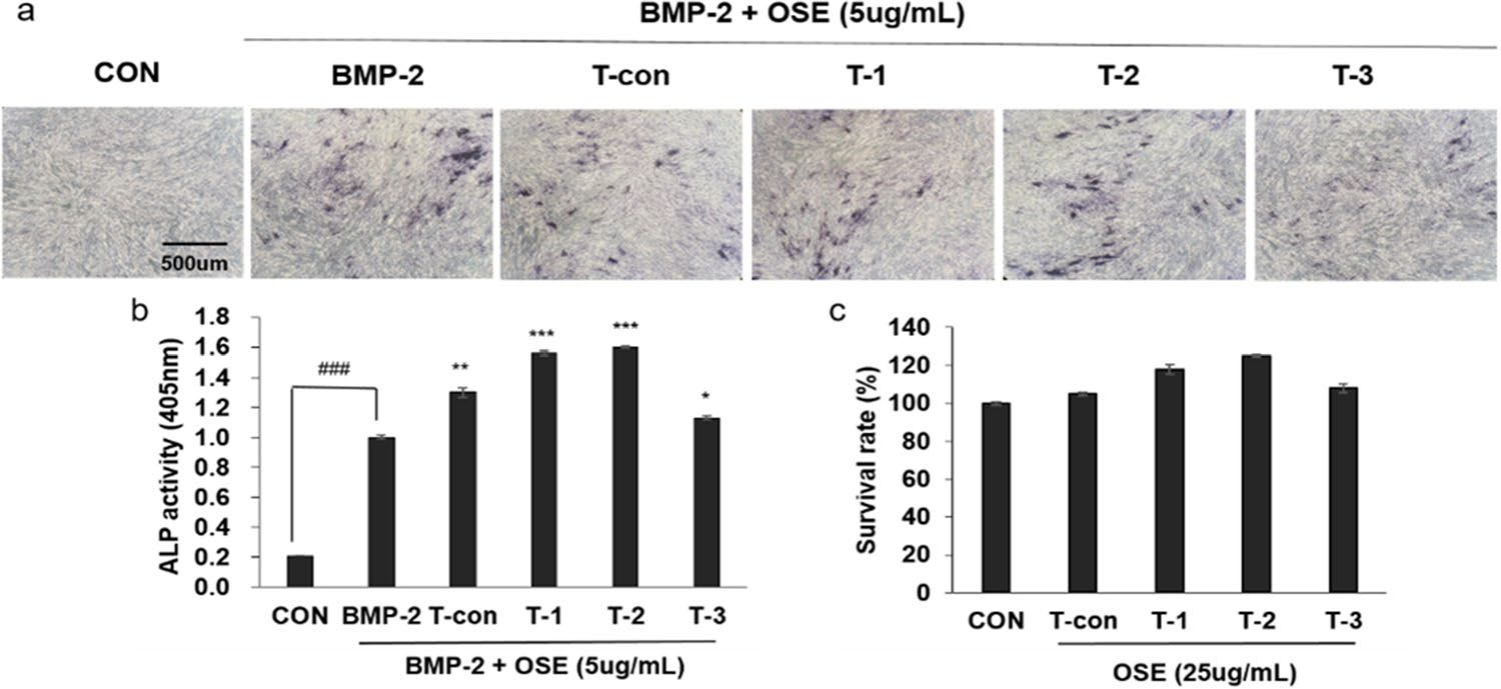
Effect of oat sprout extracts (OSE) on alkaline phosphatase (ALP) activity in C2C12 cell line. (**a**) C2C12 cells were cultured for three days in the presence of BMP-2 (20 ng/mL) with the indicated concentration of OSE. Osteoblast differentiation was visualized using ALP staining. (**b**) ALP activity was monitored by measuring the absorbance at 405 nm. ### *p* < 0.001 (vs. control); *** *p* < 0.001, ** *p* < 0.01, * *p* < 0.1 (vs. BMP-2 treated group). (**c**) Effects of OSE on the viability of C2C12 cells were evaluated using the CCK-8 assay. Each sprout was exposed to plasma for 6 min/day and ventilated as follows: T-con (the control without plasma treatment), T-1 (6 min exposure for 1 day), T-2 (6 min exposure on each day for 2 days), T-3 (6 min exposure on each day for three days)

The effect of OSE and plasma treatment on osteoblast differentiation was investigated. The ALP activity in C2C12 cells stimulated with BMP-2 was monitored after treatment with OSE, and cell viability was evaluated using a CCK-8 kit. As shown in Fig. 3, treatment with OSE at a concentration of 5 *μ*g/mL enhanced BMP-2-mediated ALP expression, which was determined using ALP staining (Fig. 3a). OSE significantly enhanced BMP-2-induced ALP activity. Plasma treatment increased the ALP expression. T-2 had the greatest effect on ALP activity (Fig. 3b). OSE had no cytotoxicity at the doses used to assess its effects (Fig. 3c). Based on the obtained results, steroidal saponins might contribute to inducing the ALP activity of OSE in C2C12 cells, and plasma treatment promoted this.

### Correlation Between ALP Activity and Metabolites

Table S2 shows the correlation between metabolites, HMGCR, pAMPK/tAMPK, and ALP activity of oat sprout extracts. ALP activity was positively correlated with avenacoside B (r = 0.911, *p* < 0.1). Policosanol and polyphenol content was not significantly correlated with ALP activity. HMGCR showed a negative correlation with isoswertisin-2″-O-rhamnoside (r = -0.908, *p* < 0.1). AMPK phosphorylation exhibit positive correlation with policosanol content(r = 0.906, *p* < 0.1) [4]. Therefore, in this study, T-1, single treated plasma was suitable for the production of oat sprouts aimed for the prevention of osteoporosis.

## Conclusions

Here, we found that cold plasma treatment during seed germination could increase the bioactive phytochemical content in oat sprouts. To our knowledge, this study is the first to investigate the effects of plasma generated by SDBD on the growth and phytochemical properties of protein expression and ALP activity in OSE. Plasma treatment affected the content of amino acids, which are the primary metabolites, and the content of secondary metabolites such as GABA, steroidal saponin, and policosanol; it also increased the physiological activities. In particular, avenacoside B content showed a positive correlation with ALP activity and was increased by plasma treatment. However, as the effect is different depending on the plasma treatment conditions, it is necessary to further investigate in animal and human models when considering raw materials for functional foods to prevent osteoblast-related bone disorders.

## Supplementary Information

Below is the link to the electronic supplementary material.

Supplementary file1 (DOCX 236 KB)

Supplementary file2 (DOCX 16 KB)

Supplementary file3 (DOCX 19 KB)

Supplementary file4 (DOCX 573 KB)

## Data Availability

The data generated in this study are available on request from the corresponding author.

## References

[1] Floros JD, Newsome R, Fisher W, Barbosa-Cánovas GV, Chen H, Dunne CP, German JB, Hall RL, Heldman DR, Karwe MV, Knabel SJ, Labuza TP, Lund DB, Newell-McGloughlin M, Robinson JL, Sebranek JG, Shewfelt RL, Tracy WF, Weaver CM, Ziegler GR (2010). Feeding the world today and tomorrow: the importance of food science and technology: an IFT scientific review. Compr Rev Food Sci Food Saf.

[2] Galili G, Höfgen R (2002). Metabolic engineering of amino acids and storage proteins in plants. Metab Eng.

[3] Batista-Silva W, Heinemann B, Rugen N, Nunes-Nesi A, Araújo WL, Braun HP, Hildebrandt TM (2019). The role of amino acid metabolism during abiotic stress release. Plant Cell Environ.

[4] Ra JE, Woo SY, Lee KS, Lee MJ, Kim HY, Ham HM, Chung IM, Kim DH, Lee JH, Seo WD (2020). Policosanol profiles and adenosine 5′-monophosphate-activated protein kinase (AMPK) activation potential of Korean wheat seedling extracts according to cultivar and growth time. Food Chem.

[5] Seo KH, Park MJ, Ra JE, Han SI, Nam MH, Kim JH, Lee JH, Seo WD (2014). Saponarin from barley sprouts inhibits NF-κβ and MAPK on LPS-induced RAW 264.7 cells. Food Funct.

[6] Pei W, Junli Y, Aaron Y, Shengmin S (2017) Avenacosides: metabolism, and potential use as exposure biomarkers of oat intake. Mol Nutr Food Res 61. 10.1002/mnfr.201700196 10.1002/mnfr.201700196 28493602

[7] Woo SY, Lee KS, Shin HL, Kim SH, Lee MJ, Young Kim HY, Ham HM, Lee DJ, Choi SW, Seo WD (2020) Two new secondary metabolites isolated from *Avena sativa* L.(oat) seedlings and their effects on osteoblast differentiation. Bioorg Med Chem Lett 30:127250. 10.1016/j.bmcl.2020.127250 10.1016/j.bmcl.2020.127250 32527550

[8] Carbonell M, Martinez E, Raya A (2000). Effects of 125 mT stationary magnetic field in the initial stages of growth of wheat. Agric Eng Res Pap.

[9] Ferrón-Carrillo F, Guil-Guerrero JL, González-Fernández MJ, Lyashenko S, Battafarano F, da Cunha-Chiamolera TPL, Urrestarazu M (2021). LED enhances plant performance and both carotenoids and nitrates profiles in lettuce. Plant Foods Hum Nutr.

[10] Šerá B, Špatenka P, Šerý M, Vrchotová N, Hrušková I (2010). Influence of plasma treatment on wheat and oat germination and early growth. IEEE Trans Plasma Sci.

[11] Song JS, Lee MJ, Ra JE, Lee KS, Eom SH, Ham HM, Kim HY, Kim SB, Lim JH (2020) Growth and bioactive phytochemicals in barley (*Hordeum vulgare* L.) sprouts affected by atmospheric pressure plasma during seed germination. J Phys D: Appl Phys 53:314002. 10.1088/1361-6463/ab810d

[12] Iimure T, Kihara M, Hirota N, Zhou T, Hayashi K, Ito K (2009). A method for production of *γ*-amino butyric acid (GABA) using barley bran supplemented with glutamate. Food Res Int.

[13] Kim SH, Kim KJ, Kang HJ, Son YJ, Choi SW, Lee MJ (2018). The dual role of oat bran water extract in bone homeostasis through the regulation of osteoclastogenesis and osteoblast differentiation. Molecules.

[14] Sivachandiran L, Khacef A (2017). Enhanced seed germination and plant growth by atmospheric pressure cold air plasma: combined effect of seed and water treatment. RSC Adv.

[15] Giorgi A, Mingozzi M, Madeo M, Speranza G, Cocucci M (2009). Effect of nitrogen starvation on the phenolic metabolism and antioxidant properties of yarrow (*Achillea collina* Becker ex Rchb.). Food Chem.

[16] Park YS, Oh KS, Oh JS, Seok DC, Kim SB, Yoo SJ, Lee MJ (2018). The biological effects of surface dielectric barrier discharge on seed germination and plant growth with barley. Plasma Processes Polym.

[17] Mitoma H, Manto M, Hampe CS (2017). Pathogenic roles of glutamic acid decarboxylase 65 autoantibodies in cerebellar ataxias. J Immunol Res.

[18] Yodpitak S, Mahatheeranont S, Boonyawan D, Sookwong P, Roytrakul S, Norkaew O (2019). Cold plasma treatment to improve germination and enhance the bioactive phytochemical content of germinated brown rice. Food Chem.

[19] Banerjee S, Ghoshal S, Porter TD (2011). Activation of AMP-kinase by policosanol requires peroxisomal metabolism. Lipids.

[20] Oliaro-Bosso S, Calcio Gaudino E, Mantegna S, Giraudo E, Meda C, Viola F, Cravotto G (2009). Regulation of HMGCoA reductase activity by policosanol and octacosadienol, a new synthetic analogue of octacosanol. Lipids.

