# Supplementary material for: Cold Plasma Treatment Increases Bioactive Metabolites in Oat (Avena sativa L.) Sprouts and Enhances In Vitro Osteogenic Activity of their Extracts
Source: Plant Foods Hum Nutr. 2022 Nov 16;78(1):146–53. doi: 10.1007/s11130-022-01029-3 (PMC9947073; doi:10.1007/s11130-022-01029-3)
Supplement: Supplementary file 1 — Supplementary file1 (DOCX 236 KB) [file 11130_2022_1029_MOESM1_ESM.docx]

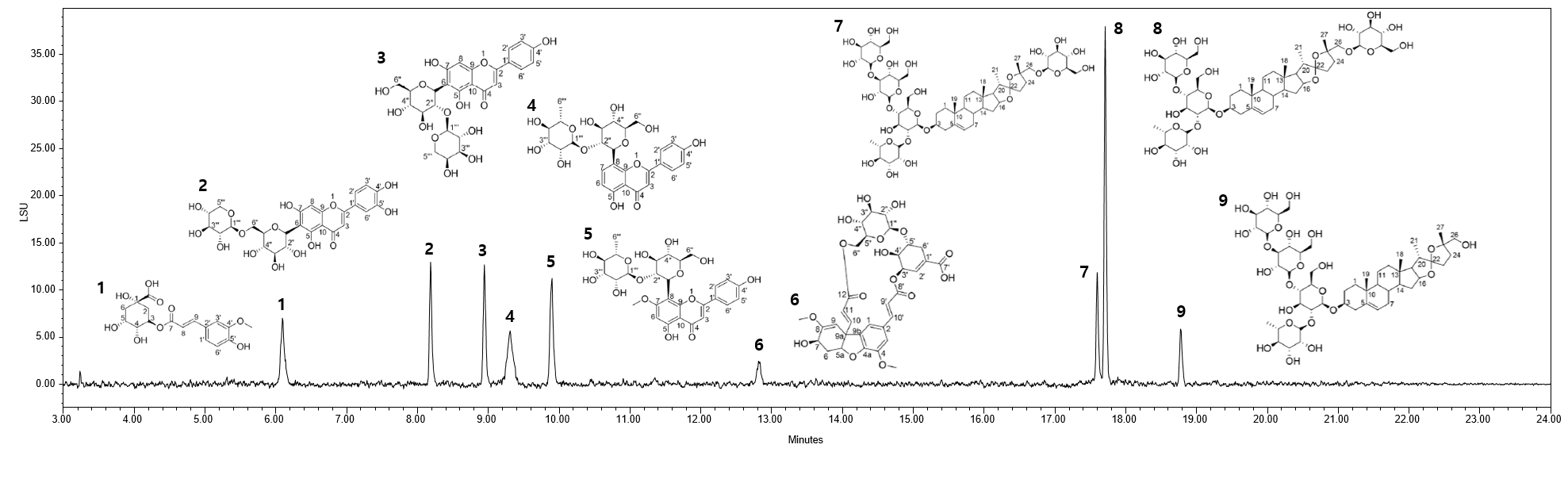
a


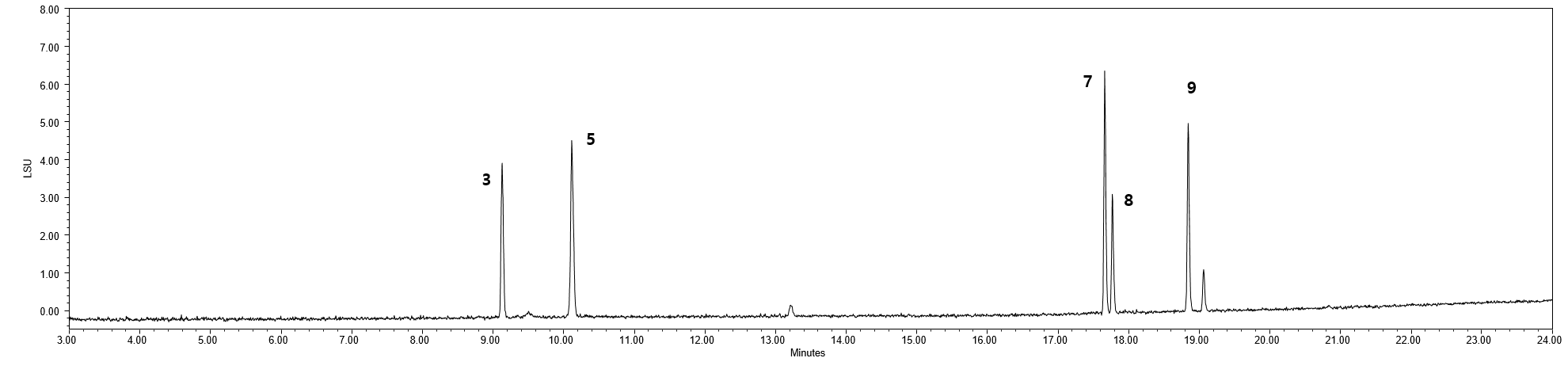
b

**Fig. S1** Chromatogram of steroidal saponins standard solution (a) and extract of T-3 oat sprouts (b).

1, 3-O-Ferubylquinic acid; 2, Isoorientin-6-β-xylopyranoside; 3, Isovitexin-2˝-o-arabinoside; 4, Vitexin-2˝-O-rhamnoside; 5, Isoswertisin-2-o-rhamnoside; 6, Avenafuranol; 7, Avenacoside b; 8, Avenacoside a; 9, 26-Deglucoavenacoside B
