# Supplementary material for: Cold Plasma Treatment Increases Bioactive Metabolites in Oat (Avena sativa L.) Sprouts and Enhances In Vitro Osteogenic Activity of their Extracts
Source: Plant Foods Hum Nutr. 2022 Nov 16;78(1):146–53. doi: 10.1007/s11130-022-01029-3 (PMC9947073; doi:10.1007/s11130-022-01029-3)
Supplement: Supplementary file 2 — Supplementary file2 (DOCX 16 KB) [file 11130_2022_1029_MOESM2_ESM.docx]

**Table S1.** Amino acid content of oat sprouts at 9 day after planting, with exposure to plasma for 6 min/day: T-con (the control without plasma treatment), T-1 (6 min exposure for 1 day); T-2 (6 min exposure on each day, for 2 days); and T-3 (6 min exposure on each day, for 3 days) during germination.

| Amino acid | Content (mg/100g) | | | |
| --- | --- | --- | --- | --- |
|  | T-con | T-1 | T-2 | T-3 |
| Histidine | 8.0±4.2 | 10.1±4.7 | 6.5±0.6 | 6.5±1.0 |
| Serine | 26.6±9.2 | 39.0±14.5 | 27.0±1.5 | 26.9±2.7 |
| Arginine | 121.2±34.3 | 169.8±49.7 | 131.3±6.0 | 96.1±6.1 |
| Glycine | 10.5±3.8 | 10.9±3.7 | 7.9±0.4 | 10.5±1.0 |
| Aspartic acid | 10.2±1.7 | 12.5±3.6 | 12.1±0.6 | 11.9±1.7 |
| Glutamic acid | 37.1±20.6 | 69.0±32.5 | 40.4±3.4 | 30.2±4.8 |
| Threonine | 16.4±7.1 | 26.0±11.7 | 16.4±1.2 | 17.6±2.6 |
| Alanine | 39.9±12.1 | 59.4±21.9 | 41.0±2.3 | 42.8±4.1 |
| Proline | 20.8±10.0 | 31.1±12.5 | 23.3±1.4 | 23.3±2.7 |
| Cysteine | 8.3±2.6 | 13.5±4.8 | 9.7±0.7 | 7.3±1.3 |
| Lysine | 22.9±11.7 | 56.2±28.6 | 32.6±2.4 | 31.4±4.6 |
| Tyrosine | 260.7±66.1 | 305.8±86.8 | 240.7±7.0 | 303.9±20.9 |
| Methionine | 14.0±6.3 | 22.2±9.5 | 14.7±1.1 | 12.7±1.7 |
| Valine | 54.1±17.0 | 77.9±27.0 | 55.7±2.7 | 53.3±4.7 |
| Isoleucine | 15.7±8.2 | 27.4±14.3 | 15.1±1.2 | 16.4±2.3 |
| Leucine | 39.1±14.4 | 57.3±22.2 | 38.5±2.1 | 40.1±4.2 |
| Phenyl alanine | 19.6±6.7 | 22.5±7.7 | 16.6±0.9 | 18.8±1.9 |
| Total | 724.9±23.6^b^ | 1010.5±12.1^a^ | 729.3±3.5^b^ | 749.5±6.7^b^ |

Data represent the mean ± standard error of triplicate experiments
