# Supplementary material for: Cold Plasma Treatment Increases Bioactive Metabolites in Oat (Avena sativa L.) Sprouts and Enhances In Vitro Osteogenic Activity of their Extracts
Source: Plant Foods Hum Nutr. 2022 Nov 16;78(1):146–53. doi: 10.1007/s11130-022-01029-3 (PMC9947073; doi:10.1007/s11130-022-01029-3)
Supplement: Supplementary file 3 — Supplementary file3 (DOCX 19 KB) [file 11130_2022_1029_MOESM3_ESM.docx]

**Table S2.** Correlation coefficients between metabolites, HMGCR, pAMPK/tAMPK, and ALP activity of oat sprout extracts.

|  | ALP | Amino acid | Poly  phenol | Poly  cosanol | Isocitexin  -2-o-arabinoside | Isoswertisin-2-o-rhamnoside | Avenaco  side B | Avenaco  side A | 26-Degluco  avenaco  side B | HMGCR | pAMPK/  tAMPK | GABA |
| --- | --- | --- | --- | --- | --- | --- | --- | --- | --- | --- | --- | --- |
| Amino acid | 0.441 |  |  |  |  |  |  |  |  |  |  |  |
| Polyphenol | -0.729 | -0.256 |  |  |  |  |  |  |  |  |  |  |
| Polycosanol | -0.729 | -0.256 | 1.000******** |  |  |  |  |  |  |  |  |  |
| Isovitexin-2-o-arabinoside | -0.994****** | -0.456 | 0.794 | 0.794 |  |  |  |  |  |  |  |  |
| Isoswertisin-2-o-rhamnoside | 0.119 | -0.652 | 0.271 | 0.271 | -0.039 |  |  |  |  |  |  |  |
| Avenacoside B | 0.911***** | 0.719 | -0.782 | -0.782 | -0.935* | -0.297 |  |  |  |  |  |  |
| Avenacoside A | 0.857 | 0.829 | -0.510 | -0.510 | -0.850 | -0.222 | 0.934 |  |  |  |  |  |
| 26-Deglucoavenacoside B | -0.933***** | -0.681 | 0.793 | 0.793 | 0.954* | 0.246 | -0.998** | -0.928* |  |  |  |  |
| HMGCR | -0.184 | 0.325 | -0.427 | -0.427 | 0.083 | -0.908* | 0.176 | -0.031 | -0.137 |  |  |  |
| pAMPK/tAMPK | -0.467 | -0.409 | 0.906* | 0.906* | 0.558 | 0.642 | -0.686 | -0.415 | 0.673 | -0.762 |  |  |
| GABA | 0.318 | 0.669 | 0.317 | 0.317 | -0.250 | 0.027 | 0.342 | 0.654 | -0.327 | -0.439 | 0.353 |  |
| Total sterol | 0.868 | 0.720 | -0.383 | -0.383 | -0.836 | 0.007 | 0.855 | 0.970* | -0.858 | -0.275 | -0.211 | 0.740 |

**p* < 0.1, ***p* < 0.01, ****p* < 0.001, *****p* < 0.0001.
