# Supplementary material for: Cold Plasma Treatment Increases Bioactive Metabolites in Oat (Avena sativa L.) Sprouts and Enhances In Vitro Osteogenic Activity of their Extracts
Source: Plant Foods Hum Nutr. 2022 Nov 16;78(1):146–53. doi: 10.1007/s11130-022-01029-3 (PMC9947073; doi:10.1007/s11130-022-01029-3)
Supplement: Supplementary file 4 — Supplementary file4 (DOCX 573 KB) [file 11130_2022_1029_MOESM4_ESM.docx]

(**b**)


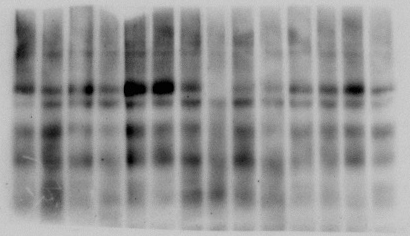


**T-con T-1 T-2 T-3**

62 kDa

p-AMPK

43 kDa

β-actin

**T-con T-1 T-2 T-3**


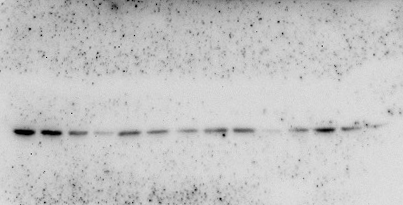


**Fig. 2** Increase in the phosphorylation of AMPK by policosanol.

AMPK phosphorylation was investigated using western blotting (**b**)

- T-con : the control without plasma treatment
- T-1 : single treatment with 6 min exposure on day 1
- T-2 : double treatment with 6 min exposure on days 1 and 2
- T-3 : triple treatment with 6 min exposure on days 1, 2, and 3
